# Convergent evolution of saccate body shapes in nematodes through distinct developmental mechanisms

**DOI:** 10.1186/s13227-019-0118-5

**Published:** 2019-03-14

**Authors:** Sita Thapa, Michael K. Gates, Ursula Reuter-Carlson, Rebecca J. Androwski, Nathan E. Schroeder

**Affiliations:** 1 Department of Crop Sciences, University of Illinois at Urbana-Champaign, Urbana, IL USA; 2 Neuroscience Program, University of Illinois at Urbana-Champaign, Urbana, IL USA

**Keywords:** Soybean cyst nematode, Root-knot nematode, Reniform nematode, Lesion nematode, *Aphelenchus*, Pyriform

## Abstract

**Background:**

The vast majority of nematode species have vermiform (worm-shaped) body plans throughout post-embryonic development. However, atypical body shapes have evolved multiple times. The plant-parasitic Tylenchomorpha nematode *Heterodera glycines* hatches as a vermiform infective juvenile. Following infection and the establishment of a feeding site, *H. glycines* grows disproportionately greater in width than length, developing into a saccate adult. Body size in *Caenorhabditis elegans* was previously shown to correlate with post-embryonic divisions of laterally positioned stem cell-like ‘seam’ cells and endoreduplication of seam cell epidermal daughters. To test if a similar mechanism produces the unusual body shape of saccate parasitic nematodes, we compared seam cell development and epidermal ploidy levels of *H. glycines* to *C. elegans*. To study the evolution of body shape development, we examined seam cell development of four additional Tylenchomorpha species with vermiform or saccate body shapes.

**Results:**

We confirmed the presence of seam cell homologs and their proliferation in *H. glycines*. This results in the adult female epidermis having approximately 1800 nuclei compared with the 139 nuclei in the primary epidermal syncytium of *C. elegans*. Similar to *C. elegans,* we found a significant correlation between *H. glycines* body volume and the number and ploidy level of epidermal nuclei. While we found that the seam cells also proliferate in the independently evolved saccate nematode *Meloidogyne incognita* following infection, the division pattern differed substantially from that seen in *H. glycines*. Interestingly, the close relative of *H. glycines, Rotylenchulus reniformis* does not undergo extensive seam cell proliferation during its development into a saccate form.

**Conclusions:**

Our data reveal that seam cell proliferation and epidermal nuclear ploidy correlate with growth in *H. glycines*. Our finding of distinct seam cell division patterns in the independently evolved saccate species *M. incognita* and *H. glycines* is suggestive of parallel evolution of saccate forms. The lack of seam cell proliferation in *R. reniformis* demonstrates that seam cell proliferation and endoreduplication are not strictly required for increased body volume and atypical body shape. We speculate that *R. reniformis* may serve as an extant transitional model for the evolution of saccate body shape.

## Background

How do body shapes evolve? Most nematodes are vermiform (i.e., worm-shaped) throughout post-embryonic development. However, several diverse nematode species develop from vermiform juveniles into saccate adult females. For example, females of the avian parasitic Tetrameridae family have a distended shape [1]. Similarly, the shark-parasitic nematode *Phlyctainophora squali* develops into a coiled and globose female [2]. Among Tylenchomorpha nematodes, several of the most economically damaging plant-parasitic nematode species develop into saccate-shaped adult females following infection [3]. *Heterodera glycines* hatches as a vermiform infective second-stage juvenile (J2). Following infection and the establishment of a feeding site, *H. glycines* females grow disproportionately greater in width than length, developing into saccate-shaped adults. Male *H. glycines* also initially grows disproportionally in width following infection. During the final juvenile stage (J4), males remodel into vermiform adults.

Not all saccate-shaped nematodes undergo the same sequence of developmental events as *H. glycines.* The closely related species *Rotylenchulus reniformis* hatches as a vermiform J2. Interestingly, *R. reniformis* does not infect following hatching, but rather molts through subsequent vermiform juvenile stages without feeding [4]. Upon molting into a vermiform adult female, *R. reniformis* infects a host and subsequently develops into a saccate-shaped female.

**Fig. 1:**
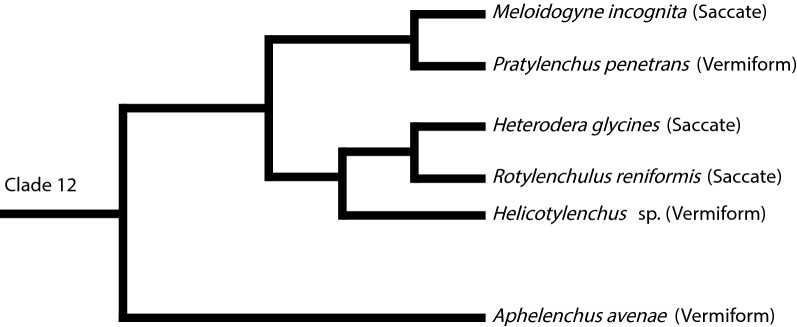
Relative phylogeny of Tylenchomorpha nematodes discussed in this study. The phylum Nematoda is divided into 12 clades [5, 6]. Tylenchomorpha nematodes are in clade 12. Branch lengths do not represent phylogenetic distance and some nodes are not consistently well supported. For example, the node separating *Heterodera* spp. and *R. reniformis* is supported by a maximum likelihood bootstrap value of 56 [6]

Based on the current hypothesized phylogeny of nematodes, development into saccate adults among Tylenchomorpha nematodes evolved at least twice (Fig. 1) [5, 6]. Similar to *H. glycines,* the independently evolved root-knot nematode, *Meloidogyne* spp. grows from a vermiform J2 into a saccate adult female following infection. Unlike *H. glycines,* much of the growth in *M. incognita* occurs prior to the molt into J3, which is quickly followed by the molts into the J4 and adult female without intervening feeding. After molting into the adult, the female resumes feeding and further growth [7]. The mechanisms regulating the development of saccate body shapes in nematodes are unknown.

**Fig. 2:**
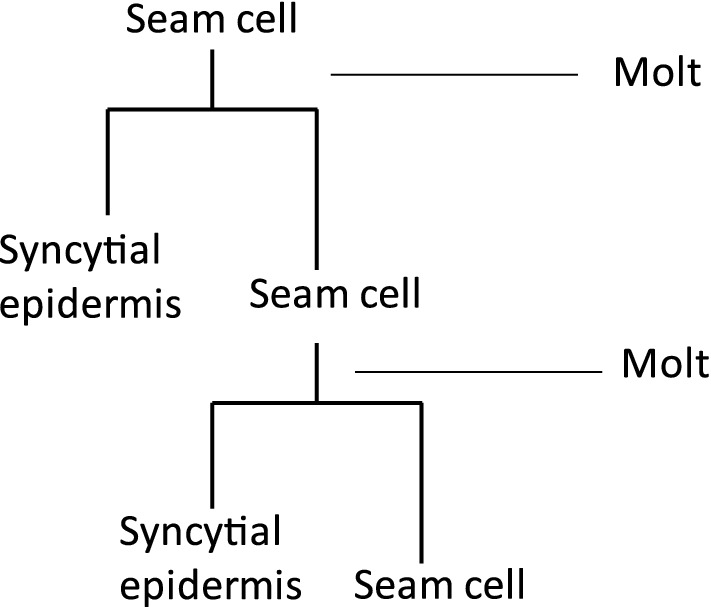
Seam cell lineage of *Caenorhabditis elegans*. Most seam cell divisions in *C. elegans* occur prior to the molt and generate a single anterior daughter cell, which fuses with the hyp7 syncytial epidermis, and a posterior daughter cell that remains a seam cell [9, 10]. Not shown are several divisions that result in neuronal or glia daughter cells

In the bacterial-feeding nematode *Caenorhabditis elegans,* the growth of the epidermis is considered a major determinant of body size [8]. Most of the *C. elegans* epidermis comprises a single large syncytium (hyp7) that grows during post-embryonic development as a succession of nuclei fuse with it [9]. The post-embryonic hyp7 nuclei are daughters of the seam cells, a series of laterally positioned epidermal cells with stem cell-like properties [10]. Newly hatched J1 *C. elegans* have ten seam cells on each lateral ridge that divide in a stem cell-like pattern before each molt (Fig. 2) [10]. Most seam cell divisions generate an anterior daughter nucleus, which fuses with hyp7, and a posterior daughter seam cell. Following the final molt, the seam cells terminally differentiate and fuse to form a separate syncytium [10, 11].

The evolution of nematode body size was suggested to be due to changes in seam cell proliferation and endoreduplication of nuclei within the epidermal syncytium [8, 12]. However, a recent report demonstrated that a newly isolated *Caenorhabditis* species evolved increased length due to increased cytoplasmic volume rather than nuclear number or ploidy [13]. We hypothesized that the *H. glycines* seam cell lineage has undergone extensive evolutionary changes to grow from a vermiform juvenile to a saccate adult female. To understand the evolution of saccate-shaped nematodes, we examined the development of *H. glycines* as well as the closely related *R. reniformis* and the independently evolved saccate species *M. incognita.* As comparisons, we also examined three Tylenchomorpha species that have typical vermiform shapes throughout development.

## Results

### The number of *H. glycines* epidermal nuclei increases following infection

**Fig. 3:**
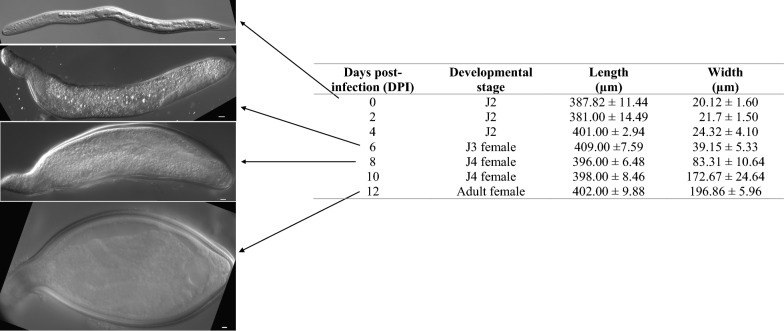
Different developmental stages of *Heterodera glycines* and size measurements. *H. glycines* hatches as second-stage vermiform juveniles (J2 s), infects the host root and initiates feeding. Following infection, their body diameter increases resulting in development from a vermiform J2 into a saccate-shaped adult female. Width measurements were made at the maximum diameter. Mean ± SEM, *n* = 3. Scale bars, 10 µm

**Fig. 4:**
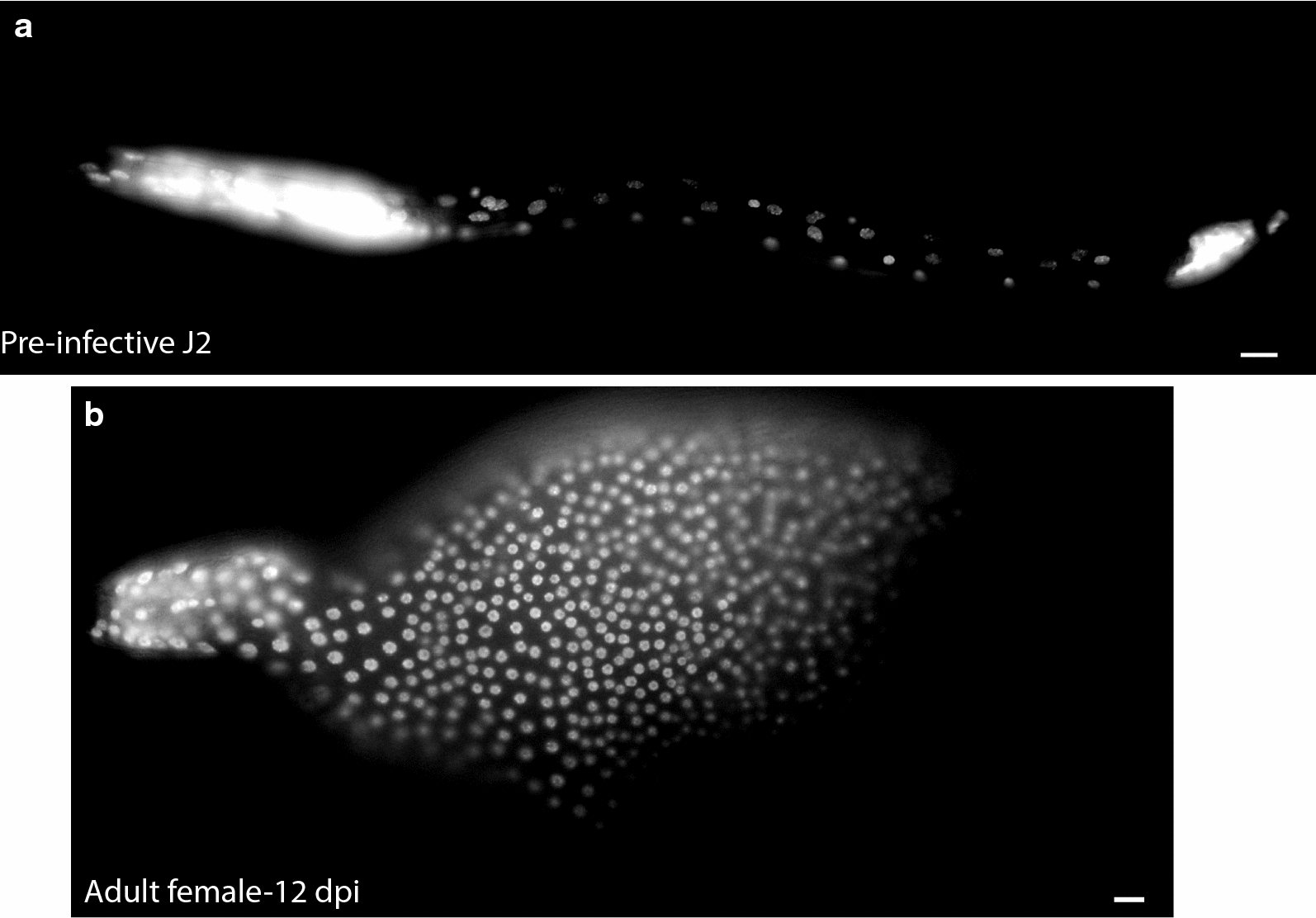
*Heterodera glycines* epidermal nuclei undergo extensive proliferation following infection. Lateral left view micrographs of DAPI-stained **a** pre-infective J2 and **b** adult female 12 days post-inoculation (DPI). Epidermal nuclei can be identified based on their shape, size, and staining intensity. Scale bars, 10 µm

*Heterodera glycines* hatches as a vermiform-shaped infective J2. Following infection, it establishes a multinucleated feeding site and develops into a saccate-shaped adult female in 11–13 days (Fig. 3). To test if the number of epidermal nuclei increases during post-hatch development, we examined DAPI-stained *H. glycines* at different developmental stages. We found 44 epidermal nuclei in pre-infective *H. glycines* J2 s (Table 1, Fig. 4a). The number of epidermal nuclei increases approximately 40-fold from the pre-infective J2 to the adult female (Table 1, Fig. 4b). In comparison, the hyp7 epidermal syncytium in *C. elegans* adult hermaphrodites comprises 139 epidermal nuclei [8].

**Table 1:** Number of epidermal nuclei in *H. glycines*

Developmental stages	No. of epidermal nuclei	Sample size	Range
Pre-infective J2	44	11	40–48
J3 female	544	7	492–656
J4 female	972	7	750–1194
Adult female	1758	6	1436–2020

### *H. glycines* has seam cell homologs

**Fig. 5:**
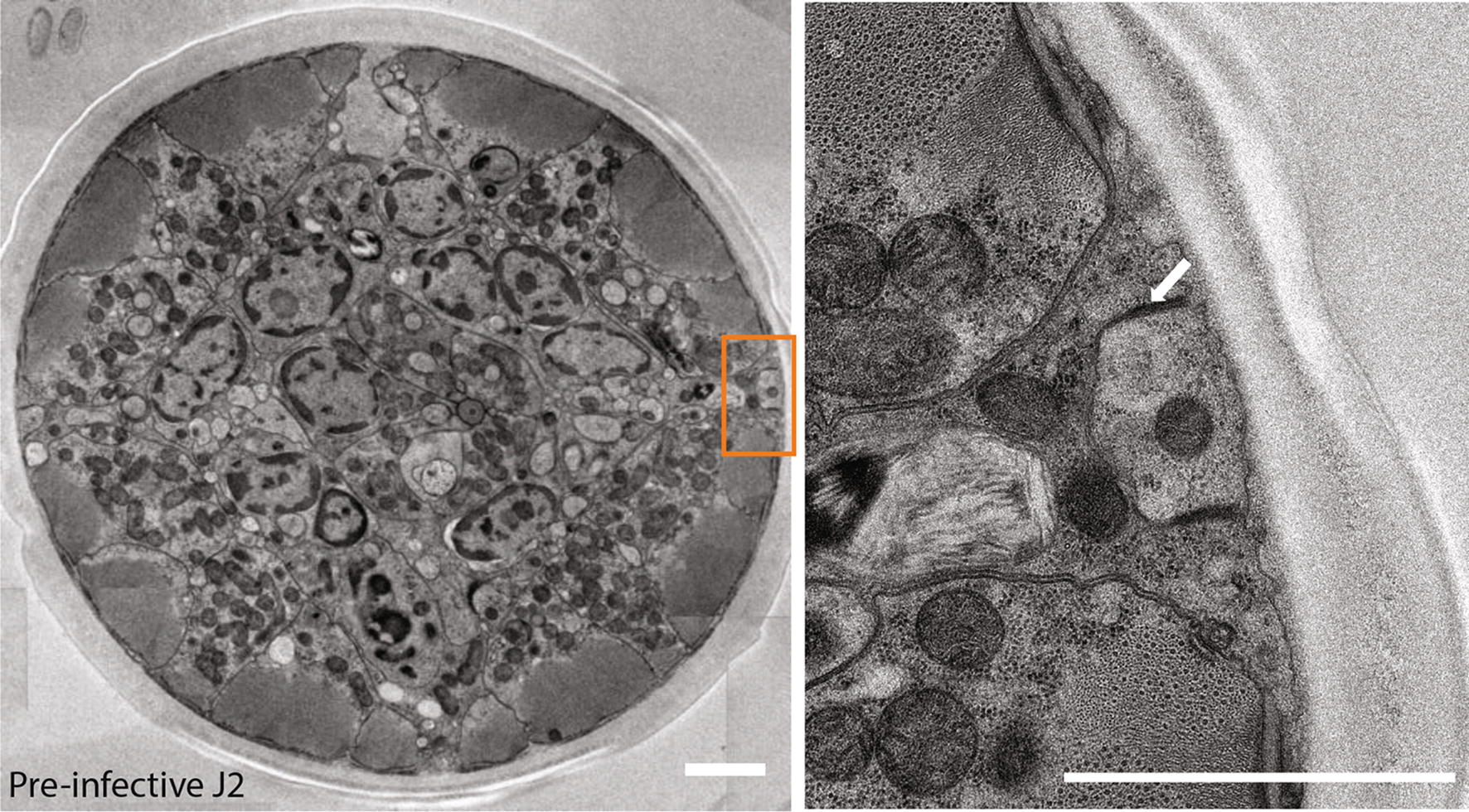
*Heterodera glycines* has seam cell homologs. Transverse TEM micrograph (left) montage through the anterior of a pre-infective J2 and (right) high-magnification image of boxed region reveal electron-dense adherens junctions (arrow) indicative of seam cells. Scale bars, 2 µm

**Fig. 6:**
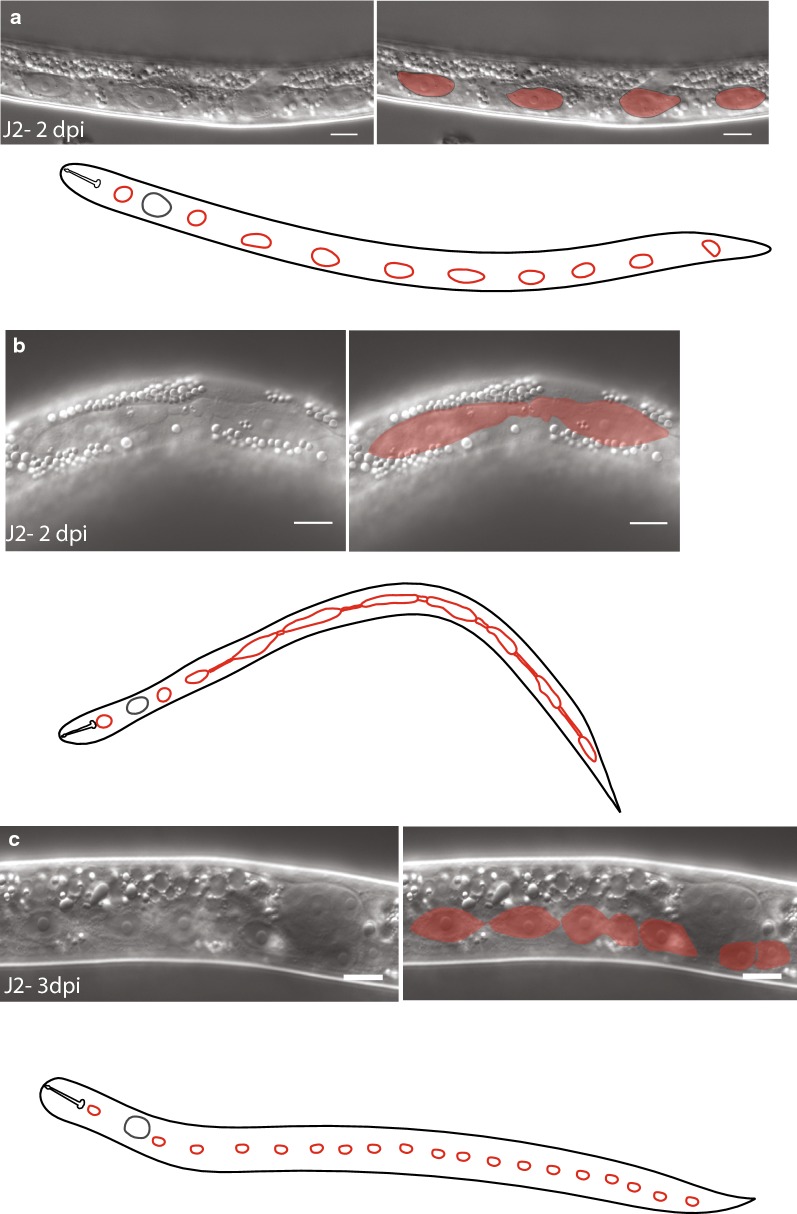
DIC micrographs of the *Heterodera glycines* lateral epidermis following infection. **a** DIC micrograph (left) and shaded overlay (right) of J2 2 days post-infection (DPI) illustrating a line of elliptical smoothly tapered seam cells along the lateral ridge. Cartoon of J2 (below) showing approximate position of seam cells (red) along the lateral epidermis. **b** DIC micrograph (left), shaded overlay (right), and cartoon schematic (below) of J2 seam cells two DPI, **c** DIC micrograph (left), shaded overlay (right), and cartoon schematic (below) of J2 seam cells three DPI, showing the change in shape and division of seam cells following infection. The timeline of individuals dissected on the same day was inferred based on examination of multiple individuals. Scale bars, 10 µm

The *C. elegans* seam cell divisions contribute to an increase in the total number of nuclei present in the surrounding syncytial epidermis (hyp7) during post-embryonic development [10]. To our knowledge, there are no data on seam cells in *H. glycines.* The *C. elegans* seam cells have a distinctive eye-shaped appearance using light microscopy with DIC optics; however, due to the highly refractile optical properties of the pre-infective *H. glycines* J2 s, we were not able to observe any seam cell-like structures at this stage. We, therefore, used transmission electron microscopy (TEM) to examine pre-infective J2 s. Similar to *C. elegans* seam cells, we found electron-dense adherens junctions on each lateral side along the length of *H. glycines* J2 s (Fig. 5) [14]. Within 2 days post-infection, the refractile optical properties of *H. glycines* are reduced and, using DIC light microscopy, we observed a line of elliptical, smoothly tapered epidermal cells along the lateral epidermis from the nose to tail (Fig. 6a). These epidermal cells are morphologically similar to *C. elegans* seam cells [10]. Similar to J1 *C. elegans*, we observed ten seam cells on each side of J2 *H. glycines.* Specifically, we found three seam cell nuclei in the head (one adjacent to the stylet, one adjacent to the metacarpus, one adjacent to the dorsal esophageal gland). Posteriorly, six seam cells lay along the length of the body and a tenth was found in the tail (Fig. 6a). Thus, using both light microscopy and TEM, we confirmed that *H. glycines* also has seam cells.

### *H. glycines* seam cells undergo extensive proliferation following infection and subsequent molts

**Fig. 7:**
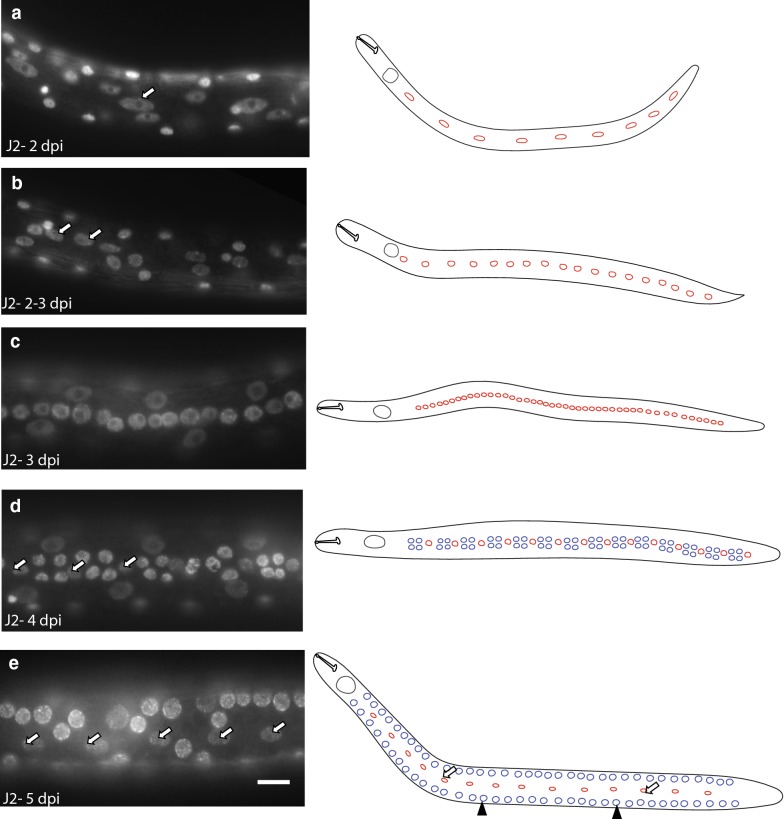
DAPI-stained fixed J2 *Heterodera glycines* demonstrates division and migration of seam cells following infection. Fluorescent micrograph (left) and cartoon schematic (right) of lateral view J2 s at multiple time points following inoculation. The timeline of individuals dissected on the same day was inferred based on examination of multiple individuals. Representative seam cells indicated with arrows. Scale bar, 10 µm

**Fig. 8:**
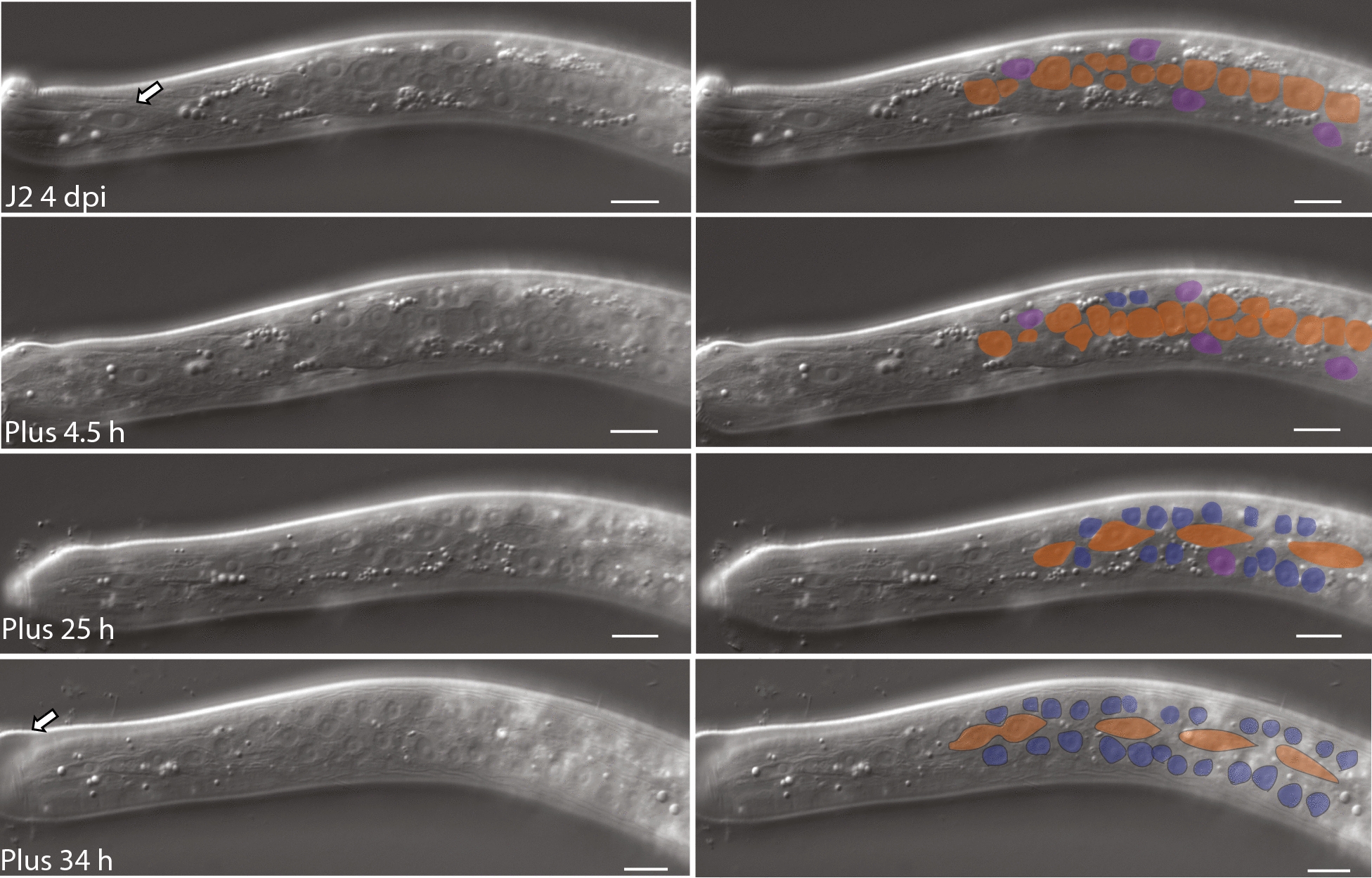
DIC micrographs of *Heterodera glycines* J2 close to the molt showing seam cell daughter cell migration. DIC micrographs (left) and shaded overlay (right) of a J2 beginning at 4 days post-infection (DPI) and subsequent time points following removal from a root. Seam cells (orange) proliferate along the lateral side posterior of the metacorpus. Prior to infection, the syncytial epidermis comprises several epidermal nuclei (purple). H0 seam cell homolog (arrow at 4 DPI) does not divide. Following proliferation, four epidermal nuclei migrate away from the lateral ridge and merge into the subdorsal and subventral epidermis (blue). Epidermal migration occurs immediately prior to the J2/J3 molt, as evidenced by the presence of the shed cuticle (arrow at 34 h). Scale bars, 10 µm

We hypothesized that growth of *H. glycines* following infection requires proliferation of the seam cells. As *H. glycines* spends much of its development within the roots of its host, we were unable to follow the cell lineage continuously. As an alternative, we dissected nematodes from the roots at multiple time points following infection and examined the seam cells in both live and DAPI-stained fixed animals. Using DIC optics, we found that following infection the posterior eight seam cells appear in contact with each other (Fig. 6b). Within 3 days following infection, the nine posterior seam cells undergo a symmetrical cell division to produce two seam cells (Figs. 6c, 7a, b). The resulting 18 seam cells undergo divisions (Fig. 7b, c), to produce four epidermal daughter cells (Fig. 7d), oriented dorsoventrally during late J2. By the time of the J2/J3 molt, we observed only elliptical-shaped nuclei (Fig. 7e arrow) along the lateral ridge suggesting an apparent dorsoventral migration of seam daughter nuclei (Fig. 7e). To confirm this migration of seam cell daughter cells from the lateral ridge, we examined the seam cell division and migration in post-infective J2 live animals dissected from the roots (Fig. 8). While plant-parasitic nematodes will not feed following removal from their host, a limited amount of development will occur. Consistent with our DAPI staining, we observed cell divisions along the lateral ridge of four dpi animals (Fig. 8). Examination of subsequent time points showed that the number of cells along the lateral line increases with time (Fig. 8, 4.5 h). As the J2 approaches the J2/J3 molt (Fig. 8, 25 h), dorsoventrally oriented cells migrated dorsoventrally (Fig. 8, 34 h). Together, our DAPI and live animal imaging suggest that proliferation of seam cells and the subsequent dorsoventral migration of daughter cells occur following infection.

**Fig. 9:**
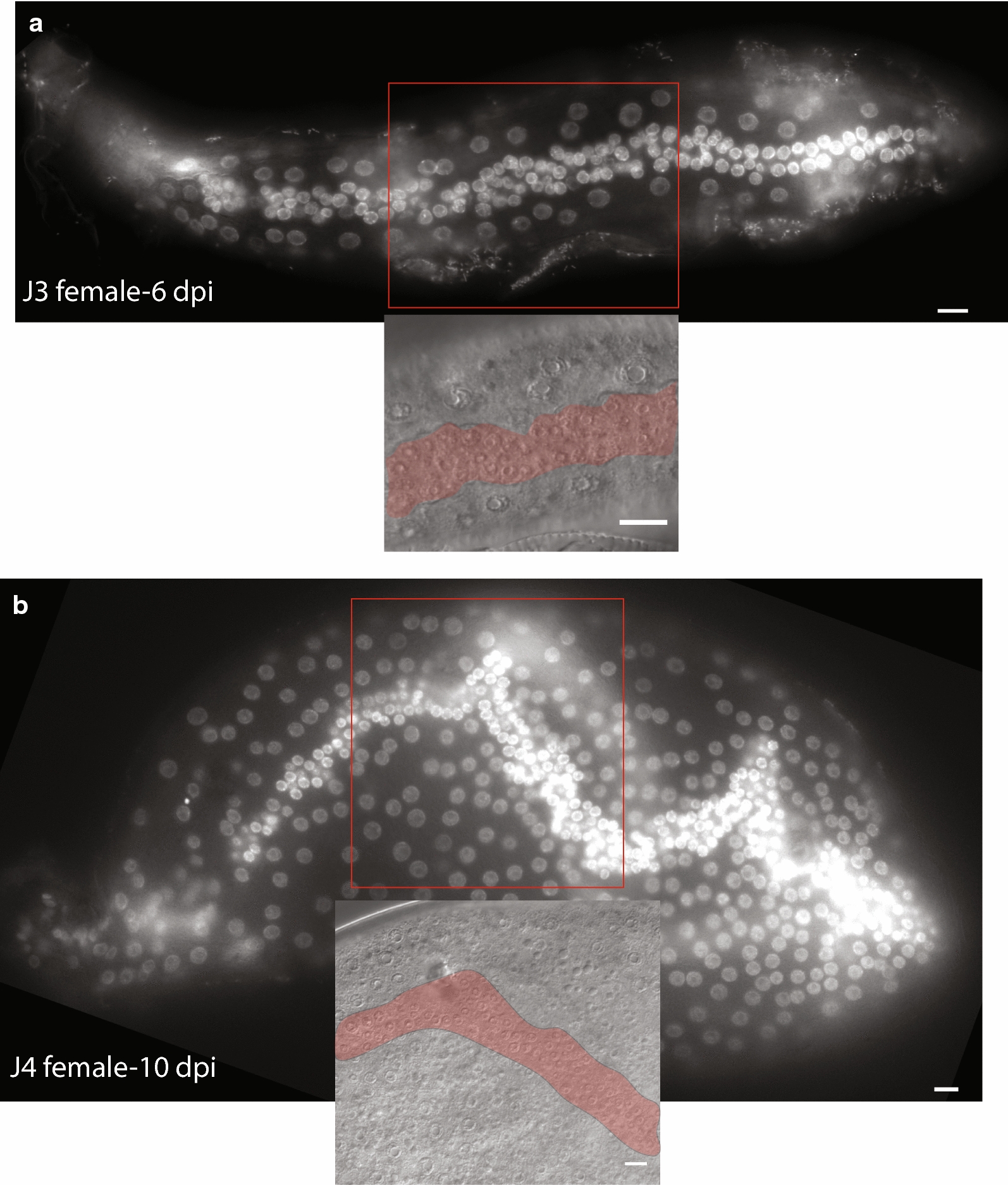
*Heterodera glycines* seam cells undergo increased proliferation at each post-infective molt lateral view micrograph of DAPI-stained nematodes showing a cluster of proliferative tissue associated with molts prior to migration into the dorsoventral sectors (compare with Fig. 3b). Insets, DIC images from same animal with pseudocolor overlay of seam cell cluster along lateral ridges. Scale bars, 10 µm

Using DAPI staining, we found that the J3/J4 and J4/adult female molts are each associated with a round of seam cell proliferation and subsequent migration of epidermal daughter nuclei away from the lateral ridge (Fig. 9). This molt-associated proliferation is similar to that seen in *C. elegans;* however, *C. elegans* seam cells do not divide during the J4/adult molt [10]. In *H. glycines,* the molts were preceded by an initial proliferation along the lateral ridge and accumulation of daughter cells that was followed by dispersal into the subdorsal and subventral regions (compare Figs. 4b, 8). Interestingly, each subsequent molt appeared to result in the production of more daughter cells.

**Fig. 10:**
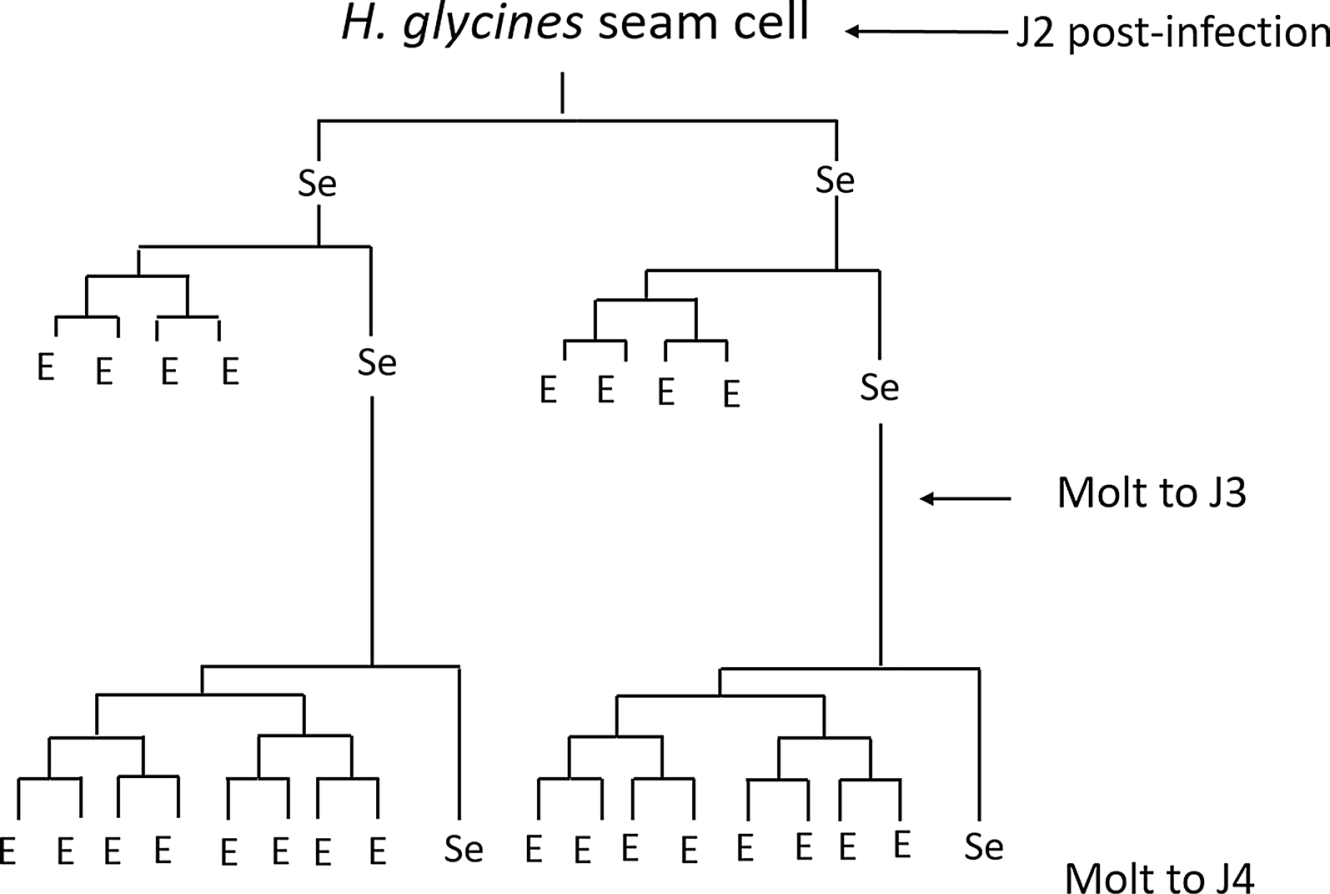
Working model of *Heterodera glycines* female seam cell division post-infection. Based on observations at multiple time points, we propose that *H. glycines* J2 seam cells divide following infection to produce two daughter seam cells, which then undergo additional divisions to produce four epidermal nuclei and one seam cell. Subsequent molts result in an increased number of divisions and resulting epidermal nuclei. The number of epidermal nuclei produced by each seam cell was estimated based on the total number of epidermal nuclei found after each molt; however, due to the obligate endoparasitic lifestyle of *H. glycines,* we are unable to confirm the precise lineage of individual cells

The proliferation of seam cells during the J3/J4 and J4/adult molts did not occur in a perfectly synchronous pattern within individual nematodes. This asynchrony combined with the difficulties in synchronizing infection made it impossible to quantify the precise number and lineage of daughter nuclei produced by each seam cell at these later time points. We found that *H. glycines* J3 and J4 females have 544 and 972 epidermal nuclei, respectively (Table 1). Each successive molt resulted in an approximate doubling of epidermal nuclei (Table 1). After the final molt, an average of 1758 epidermal nuclei were evenly distributed in adult females (Table 1 and Fig. 4b). While we cannot be confident in the precise pattern of divisions, our findings suggest a possible lineage model where the number of divisions increases at each successive molt (Fig. 10).

### *H. glycines* has an epidermal organization similar to *C. elegans*

**Fig. 11:**
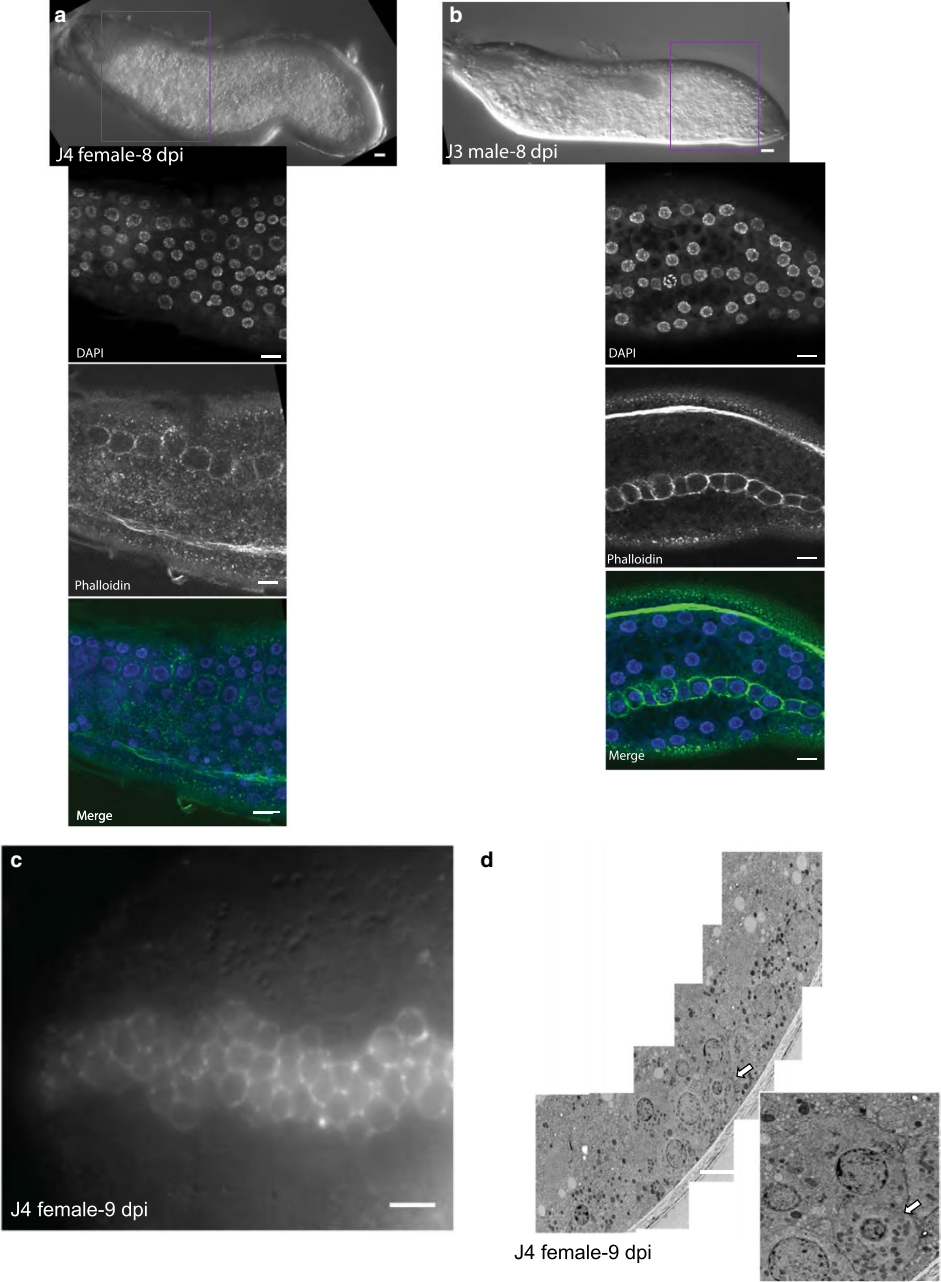
*Heterodera glycines* epidermis is a syncytium. **a** DIC (top) and fluorescent micrographs (insets) of an 8 day post-infection (DPI) J4 female following migration of epidermal nuclei and stained with both phalloidin and DAPI. At this stage, the seam cells are a single line of cells on the lateral ridge. Phalloidin fluorescence was observed surrounding each nucleus within the lateral seam, but not in the surrounding epidermis. **b** DIC (top) and fluorescent micrographs (insets) of an eight DPI J3 male. Phalloidin fluorescence was observed surrounding each nucleus within the lateral seam, but not in the surrounding epidermis. DAPI labels nuclei throughout the epidermis. **c** Fluorescent micrographs of phalloidin-stained nine DPI female following proliferation, but prior to migration of daughter epidermal cells within the lateral ridge. Scale bars, 10 µm. **d** TEM micrograph of nine DPI females with a cluster of nuclei on the lateral margins with distinct cell membranes. Inset, magnified image of lateral nuclei showing cell membranes (arrows) Scale bars, 200 nm

Most of the post-embryonic *C. elegans* epidermis comprises a multinucleate syncytium that surrounds the lateral seam cells [15]. To determine if the *H. glycines* epidermis has a similar organization, we stained post-infective nematodes with the F-actin binding fluorescent probe AlexaFluor phalloidin. In phalloidin-stained animals, we observed bright fluorescence surrounding each nucleus within the lateral seam, but none in the surrounding epidermis suggesting a similar epidermal syncytial organization to *C. elegans* (Fig. 11a, c). To confirm this observation, we examined post-infective juveniles with TEM and found a cluster of nuclei on the lateral ridge with distinct cell membranes (Fig. 11d). This cluster of seam cells was surrounded by a larger epidermal area with periodic nuclei not interrupted with apparent cell membranes (Fig. 11d). Together, these data suggest that *H. glycines* seam cells are surrounded by an epidermal syncytium. Recent research showed that the *C. elegans* derived antibody MH27 labels apical junctions in the Tylenchina nematode *Zeldia punctata* [16]. Unfortunately, we were not successful in our attempts at staining *H. glycines* with MH27.

### *H. glycines* male seam cells proliferate less than female seam cells

**Fig. 12:**
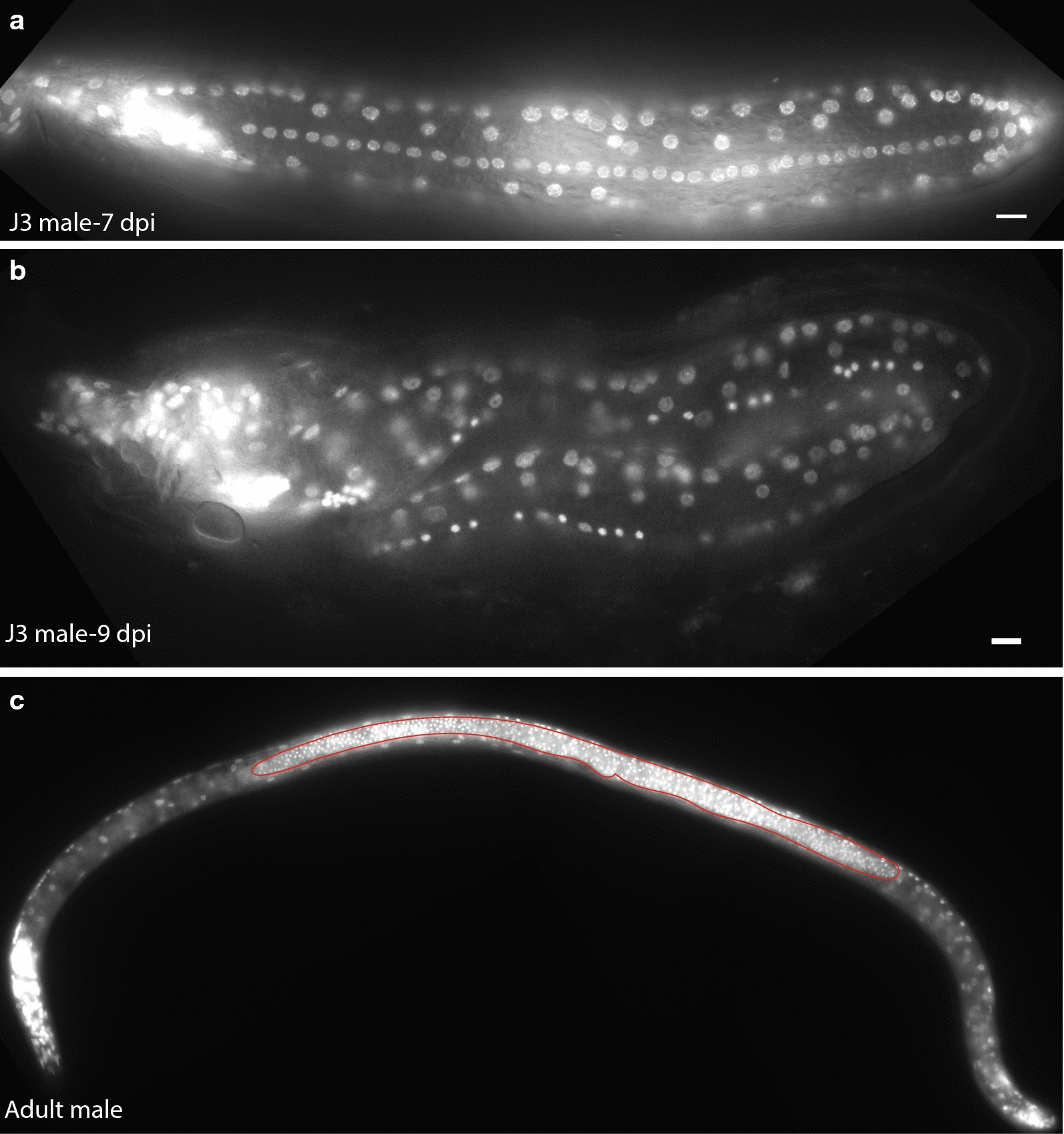
*Heterodera glycines* male seam cells proliferate less than females. **a** Lateral view fluorescent micrograph of DAPI-stained J3 male 7 days post-inoculation (DPI). Nuclei along the lateral side are produced during the J3 stage through seam cell division. **b** J4 males are highly coiled within the old J3 cuticle, making an examination of seam cells difficult. (C) DAPI-stained fluorescent micrograph of an adult male with bright fluorescence from the sperm nuclei (outlined in red), making it difficult to quantify epidermal nuclei. Scale bars, 10 µm

Similar to J3 females, *H. glycines* J3 males are sausage-shaped. Using phalloidin, we found that *H. glycines* male seam cells are also surrounded by a syncytium (Fig. 11b). Interestingly, we found that *H. glycines* J3 male seam cells proliferate less than in females (Fig. 12a, compared to 9a). Unlike in J3 females, where seam daughter nuclei migrate dorsoventrally, in *H. glycines* J3 males, they remain in the lateral epidermis (Fig. 12a). By the J3/J4 molt, *H. glycines* males begin remodeling back to a vermiform where they become coiled within the old cuticle. Due to the coiling of male J4 s (Fig. 12b) and background fluorescence from sperm cells in adult males (Fig. 12c), we were unable to accurately quantify the number of epidermal nuclei. Similarly, in live adult male animals, we were not able to accurately count the number of epidermal nuclei due to the increased refractile nature for live DIC imaging and the tendency of adult male *H. glycines* to twist along their longitudinal axis.

### *H. glycines* epidermal nuclei are polyploid

**Fig. 13:**
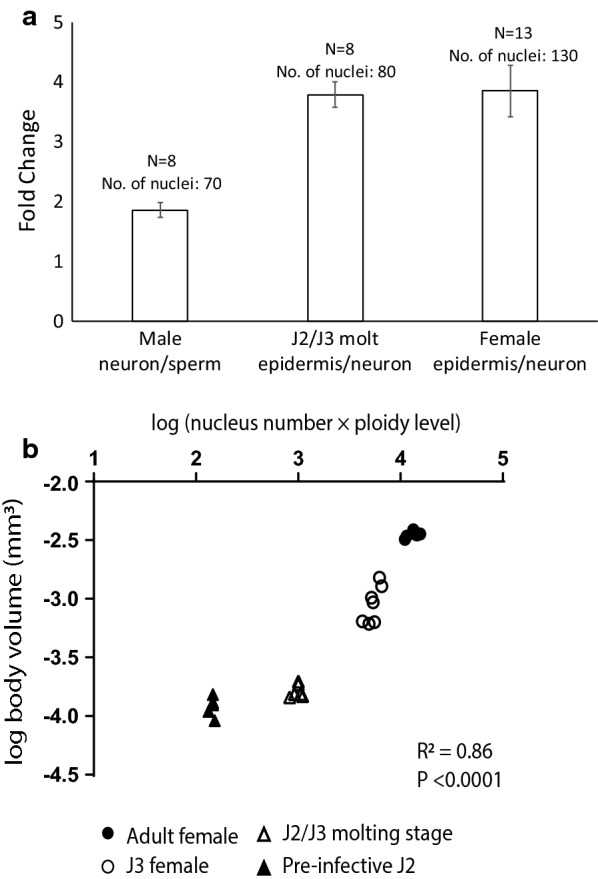
*Heterodera glycines* epidermal proliferation and polyploidy correlate with body size during development. **a** *H. glycines* epidermal nuclei are polyploid as determined by microdensitometry. As expected, we found an approximate doubling of fluorescence intensity between the haploid sperm and diploid neurons. The fluorescent intensity of epidermal nuclei was 3.55 and 3.85 times greater than neuronal nuclei in J2/J3 molting nematodes and adult females, respectively. This suggests an epidermal ploidy of 7.1C (C being haploid DNA) for J2/J3 s and 7.7C for adult females. **b** A significant correlation exists between the estimated body volume and the product of the number of epidermal nuclei and the ploidy level at different developmental stages (*R*^2^ = 0.86; *P* < 0.0001). Each point represents a single nematode. The number of epidermal nuclei counted on one side was doubled to estimate the total number of epidermal nuclei

*Caenorhabditis elegans* epidermal nuclei are polyploid [17] and previous results showed a correlation between body volume and the product of epidermal ploidy level and the number of epidermal nuclei among several bacterial-feeding nematodes [8]. We measured the DNA content of *H. glycines* epidermal nuclei during the J2/J3 molt and adult females using microdensitometry. To confirm our intensity measurements, we first compared haploid sperm nuclei fluorescent intensity to diploid neuronal nuclei. As expected, we found an approximate doubling of fluorescence between the haploid sperm and diploid neurons (Fig. 13a). Fluorescent intensity was then compared between female neuronal nuclei and epidermal nuclei at the J2/J3 molt and in adult females. We observed a threefold to fourfold increase in fluorescent intensity between diploid neuronal nuclei and epidermal nuclei, suggestive of epidermal polyploidy (7.1C in J2/J3, 7.7C in adult) (Fig. 13a). While there was substantial variation among individuals, overall our data suggest that *H. glycines* epidermal nuclei are polyploid.

### *H. glycines* number and ploidy level of epidermal nuclei correlate with body size

We conducted a regression analysis to determine if the increasing number of epidermal nuclei and ploidy level at each successive molt correlate with the increasing body size of *H. glycines*. We found a significant correlation between the product of number and ploidy level of epidermal nuclei and the estimated body volume (*R*^2^ = 0.86; *P* < 0.0001) of *H. glycines* at different developmental stages (Fig. 13b). Our results demonstrate that the growth of *H. glycines* from a vermiform J2 to a saccate adult female is associated with extensive proliferation of the seam cells and polyploidy.

**Fig. 14:**
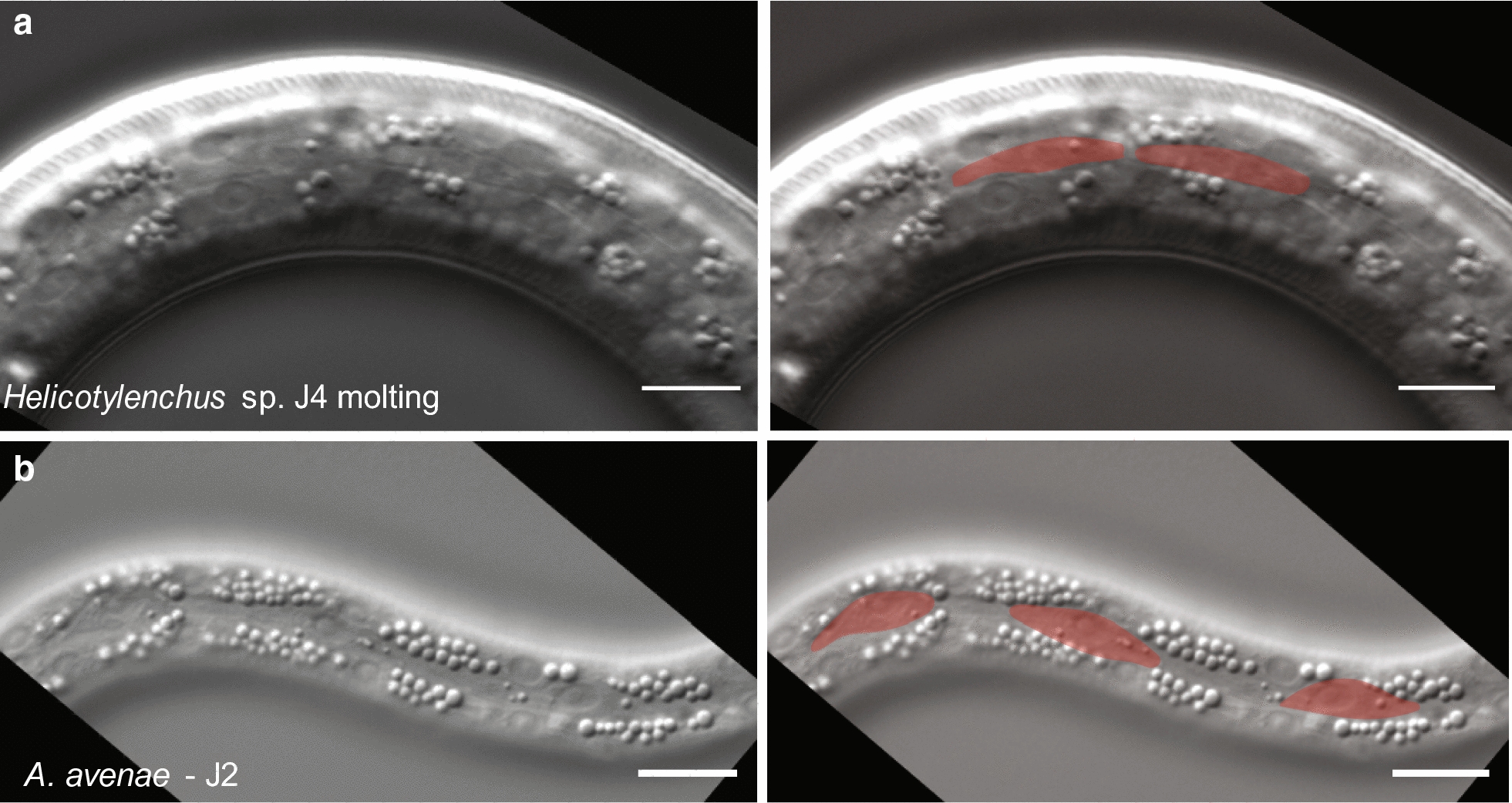
*Helicotylenchus* sp. and *Aphelenchus avenae* have seam cell homologs. Lateral view DIC micrographs (left) and pseudocolor overlay (right) of **a** J4 molting stage *Helicotylenchus* sp. and **b** J2 stage *A. avenae*. In both species, seam cells were morphologically similar to *C. elegans* [10]. Scale bars, 10 µm

Because there was a large difference in the number of epidermal nuclei between *H. glycines* and previously examined vermiform bacterial feeders [8], we were curious whether this was due to a clade-wide change in the relationship of epidermal nuclei and ploidy to body size. We, therefore, examined the number and ploidy of epidermal nuclei in vermiform Tylenchomorpha species. Hoplolaimidae (*Helicotylenchus* sp.) is a sister family to *H. glycines* Heteroderidae (Fig. 1); however, *Helicotylenchus* sp. is vermiform throughout post-embryonic development. We observed seam cells along the lateral ridge of *Helicotylenchus* sp., morphologically similar to *H. glycines* and *C. elegans* (Fig. 14a), [10]. We found that J4 female *Helicotylenchus* sp. contained 194 epidermal nuclei (*n* = 13) with a mean ploidy of 3.4C (*n* = 13; number of nuclei = 130). We also examined *Aphelenchus avenae*, a fungal feeding vermiform Tylenchomorpha (Fig. 1). Again, we observed seam cells along the lateral ridge (Fig. 14b) [10]. In *A. avenae* J4 females, we found 220 epidermal nuclei (*n* = 6) and a ploidy of 3.3C (*n* = 6; number of nuclei = 60). Together, these data suggest that the epidermal proliferation of *H. glycines* was not due to an earlier evolutionary event leading to a general increase in epidermal nuclei in all Tylenchomorpha.

### Divergent division patterns acting on conserved seam cells gave rise to independently evolved saccate body shapes

**Fig. 15:**
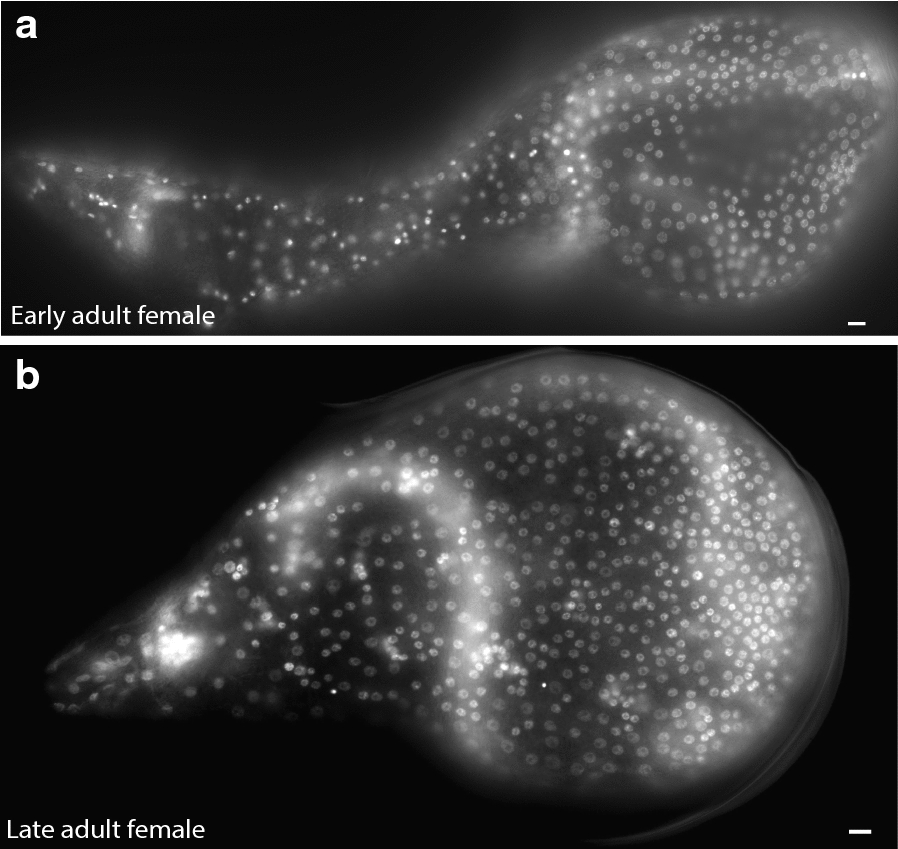
*Meloidogyne incognita* females undergo continued epidermal proliferation in adulthood **a** fluorescent micrograph of DAPI-stained young adult female with approximately 600 epidermal nuclei. **b** Late adult female with approximately 950 epidermal nuclei. Scale bars, 10 µm

Saccate body shapes likely evolved multiple times among nematodes (Fig. 1) [5, 6]. To determine if the saccate shape in other nematodes is also correlated with seam cell proliferation, we examined the independently evolved saccate nematode *M. incognita* (Fig. 1). We hypothesized that despite their independent origins, *H. glycines* and *M. incognita* would use a similar pattern of seam cell proliferation to increase the number of epidermal nuclei and ploidy level associated with their increased volumes. To determine if the number and ploidy of epidermal nuclei is associated with an independently derived saccate body form, we examined the young adult females of *M. incognita*. We counted 598 epidermal nuclei (*n* = 9) and a mean ploidy level of 4.67C (*n* = 9, no of nuclei = 90) in *M. incognita*. Young adult *M. incognita* females continue to grow even after the final molt, and our preliminary data suggest a corresponding increase in the number of epidermal nuclei; late adult females have 948 epidermal nuclei (*n* = 2) (Fig. 15). As a closely related vermiform control (Fig. 1), we found that *Pratylenchus penetrans* J4 females have an average 100 epidermal nuclei (*n* = 5). Due to background fluorescence in *P. penetrans,* we were unable to accurately measure the ploidy level in this species. Our results suggest that similar to *H. glycines*, *M. incognita* proliferation of epidermal nuclei is associated with a saccate body shape.

**Fig. 16:**
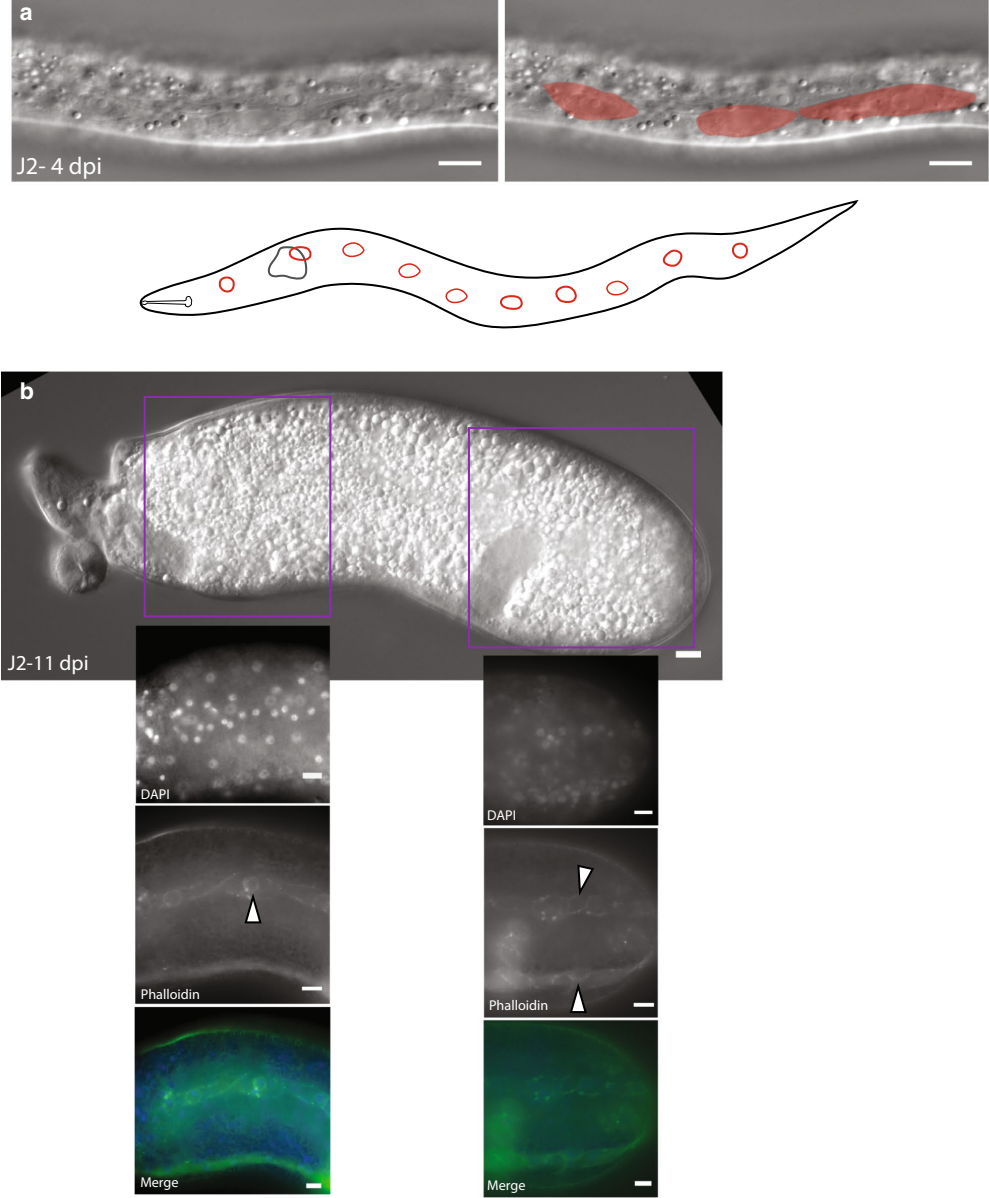
*Meloidogyne incognita* uses seam cell proliferation during development, but with a different pattern of division. **a** Lateral view DIC micrograph (left), pseudocolor overlay (right), and cartoon schematic (below) of a J2 *M. incognita* 4 days post-infection (DPI) with a line of 10 elliptical smoothly tapered epidermal cells along the lateral ridge characteristic of seam cells. **b** DIC (top) and fluorescent micrographs (insets) of 11 DPI J2 *M. incognita* stained with phalloidin and DAPI. Phalloidin staining revealed putative apical junctions with a pattern distinct from that seen in *H. glycines*. Scale bars, 10 µm

Unlike *H. glycines,* which grows throughout each juvenile stage, much of the growth in *Meloidogyne* spp. occurs during J2 following infection with very little increase in size occurring during the J3 and J4 stages [7]. We found that J2 *M. incognita* extracted soon after infection have a line of epidermal cells along the lateral seam morphologically similar to *H. glycines* and *C. elegans* seam cells (Fig. 16a) [10]. To further examine the proliferation of epidermal nuclei in *M. incognita,* we conducted an analysis of DAPI-stained juveniles at successive time points following infection. Following the J2/J3 molt, we did not observe a clustering of nuclei along the lateral ridge as observed during *H. glycines.* Interestingly, staining with phalloidin revealed putative cell junctions in the subventral and subdorsal quadrants and an unusual pattern of staining along the lateral seam (Fig. 16b). While our time points are insufficient to propose a comprehensive model of epidermal division in *M. incognita,* the pattern of divisions appears to deviate from *H. glycines*.

### *R. reniformis* does not use seam cell proliferation to increase body size

**Fig. 17:**
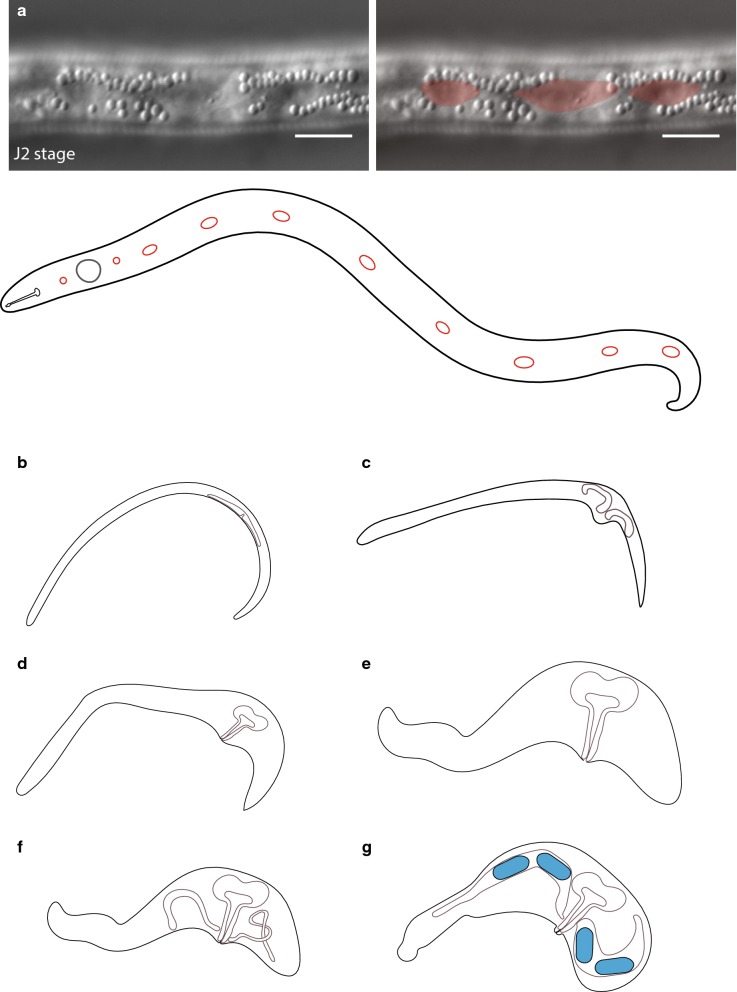
*Rotylenchulus reniformis* seam cells do not proliferate following infection. **a** Lateral view DIC micrograph (left), pseudocolor overlay (right), and cartoon schematic (below) of J2 *R. reniformis* with elliptical smoothly tapered cells along the lateral ridge characteristic of seam cells. Scale bars, 10 µm. **b**–**g** Cartoon schematic of *R. reniformis* adult females prior to (**b**) and during (**c**–**g**) infection

*Rotylenchulus reniformis* is a saccate-shaped nematode and phylogenetically proximal to *H. glycines* (Fig. 1). Unlike *H. glycines,* the adult female of *R. reniformis* is the infective stage and becomes saccate following infection. The mature saccate *R. reniformis* female is smaller in volume compared with *H. glycines*. We, therefore, hypothesized that *R. reniformis* would have an intermediate number of nuclei between *H. glycines* and *Helicotylenchus* sp. Similar to *H. glycines* and *C. elegans,* we found a line of laterally positioned epidermal nuclei consistent with seam cells (Fig. 17a) [10]. The *R. reniformis* adult parasitic female has only 50 epidermal nuclei (*n* = 8) and a ploidy level of 5.2C (*n* = 8; number of nuclei = 80). We conducted a time-course analysis, but did not observe any epidermal divisions following infection. Rather, we observed that swelling of the body occurred adjacent to and concurrent with the enlarging gonad (Fig. 17b–g). This result suggests that epidermal proliferation is not strictly required for the development of a saccate body shape.

### Atypical body shape in nematodes is not strictly associated with the number or ploidy level of epidermal nuclei

**Fig. 18:**
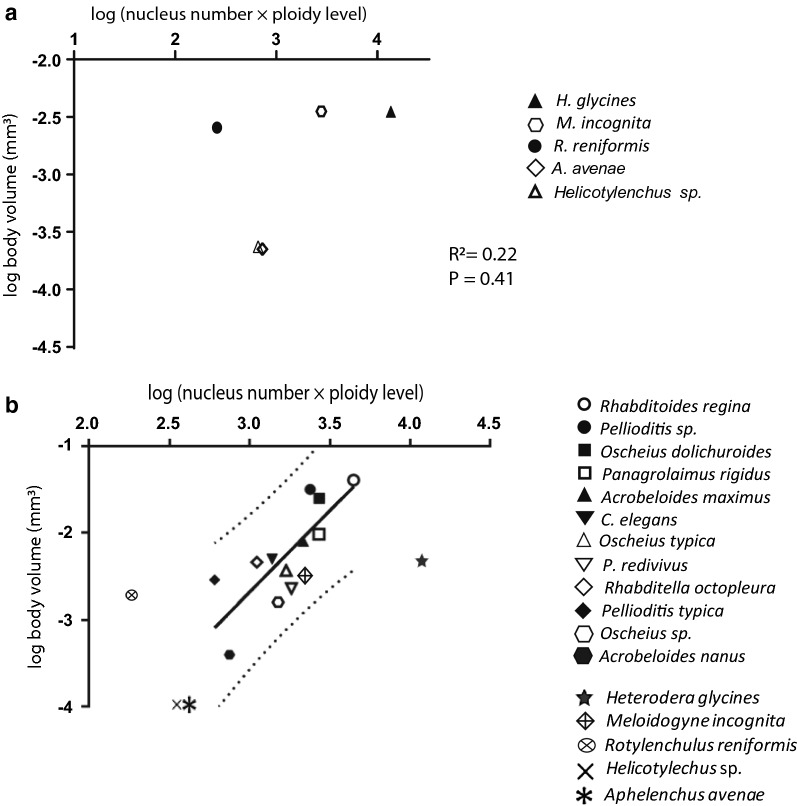
Atypical body shape in nematodes is not strictly associated with the number or ploidy level of epidermal nuclei. **a** There is no significant association between estimated body volume and the product of number of epidermal nuclei and ploidy level across Tylenchomorpha species (*R*^2^ = 0.22; *P* = 0.41). **b** The body volume and the product of epidermal nuclear number and ploidy from Tylenchomorpha nematodes were plotted onto the regression line from Flemming et al. [8] with 95% prediction intervals (dotted curves). We found that *H. glycines* and *R. reniformis* lay outside of the 95% prediction interval

We examined the relationship between the body volume and the number and ploidy level of epidermal nuclei across species (*H. glycines*, *M. incognita*, *R. reniformis*, *A. avenae*, and *Helicotylenchus* sp.). We found no significant association between the estimated body volume and the product of number and ploidy of epidermal nuclei across species (*R*^2^ = 0.22; *P* = 0.41), (Fig. 18a). This finding, while contradictory to Flemming et al. [8], is in agreement with recent results showing a large size difference between *C. elegans* and a newly described *Caenorhabditis* sp. that does not correlate with changes in epidermal nuclear number or ploidy [13]. To further examine the relationship between body size and epidermal nuclear number/ploidy, we mapped our data from Tylenchomorpha species onto the linear regression model from Flemming et al. [8]. Interestingly, we found *H. glycines* and *R. reniformis* lay outside of the 95% prediction interval of their data (Fig. 18b).

## Discussion

Our results suggest that the stem cell-like seam cells are conserved in Tylenchomorpha nematodes. Similar to *C. elegans,* we found that the lateral ridge of *H. glycines* comprises elliptical-shaped cells with proliferative capacity. Furthermore, using TEM, we observed characteristic adherens junctions along the lateral ridge. Using light microscopy, we found similar cells in all nematodes examined here. While we did not conduct TEM on other species, an examination of published and unpublished TEM data suggests that adherens junctions along the lateral ridge are also present in *Meloidogyne incognita* and *Pratylenchus penetrans* [18, 19]. Seam cells are present in bacterial-feeding nematodes *Panagrellus redivivus* and *Pristionchus pacificus* [20, 21]. Similarly, the seam cells were found in a group of diverse bacterial-feeding nematodes [8]. We hypothesize that seam cells are conserved within the crown clades of Chromadoria [5, 6].

We show that epidermal proliferation is extensive in *H. glycines.* Most *C. elegans* seam cell divisions lead to the formation of a single hyp7 epidermal nucleus and a posterior seam cell [10]. In contrast, we found that seam cell proliferation in *H. glycines* leads to increasing numbers of epidermal nuclei daughter cells at each successive molt (Fig. 10). Occasionally, the *C. elegans* seam lineage leads to the formation of glia or neuronal daughters. For example, the *C. elegans* seam cell V5 leads to the formation of the post-deirid neurons prior to the L2 molt. Due to the parasitic nature of *H. glycines* development, we were unable to follow the precise lineage of seam cells; however, we did not observe DAPI-stained nuclei with a neuron-like morphology and a position suggestive of post-deirids.

Our results may suggest that epidermal proliferation and the increase in epidermal ploidy cause the increase in body volume of *H. glycines*. We found a strong correlation between the product of epidermal nuclear number by ploidy and the body volume. Interestingly, we also found that the marked sexual dimorphism in *H. glycines* is represented by a different number of epidermal nuclei found during the J3 stage. These results support a possible causative link between epidermal nuclei and body size. The primary control strategy for *H. glycines* is host resistance. *H. glycines* females are smaller on resistant varieties than on susceptible varieties [22]. It will be interesting to know if this size difference is due to decreased seam cell proliferation or epidermal ploidy.

A question our data still do not answer is why epidermal proliferation in *H. glycines* does not simply lead to large vermiform nematodes that grow proportionally in length and width? In *C. elegans*, the elongation of embryos beyond the twofold stage requires the contraction of body wall muscles [23]. Ablation of *C. elegans* body wall muscle leads to swelling around the destroyed cells [24]. We recently demonstrated that *H. glycines* and *M. incognita* undergo progressive muscle atrophy with the onset of the sedentary life cycle [25]. This atrophy corresponds with the increase in size. We speculate that in *H. glycines* and *M. incognita,* the atrophy of body wall muscles following infection prevents the maintenance of a vermiform shape by eliminating the lengthening forces found in other nematodes.

Body size in nematodes was hypothesized to have evolved due to changes in the number and ploidy of epidermal nuclei [8]. Our data do not strictly fit that model. While we found a significant correlation between the product of the number and ploidy of epidermal nuclei and *H. glycines* body size during post-embryonic development; our *H. glycines* data lay outside of the 95% prediction interval proposed by Flemming et al. [8]. Indeed, based on the number and ploidy of epidermal nuclei in adult *H. glycines* females, the Flemming model predicts a body volume 100-fold greater than our experimental measurements. We previously showed that the *H. glycines* epidermis increases in thickness following infection [25]. Also, post-infective *H. glycines* secretes exudates from the cuticle that are proposed to originate from the thickened epidermis [26]. Perhaps, rather than increased body volume, the unexpectedly large number of epidermal nuclei and increased ploidy in *H. glycines* are used to provide increased epidermal thickness and protein synthesis.

Our results suggest that multiple mechanisms may lead to a saccate body shape. Similar to *H. glycines*, *M. incognita* undergoes extensive epidermal proliferation and epidermal polyploidy. However, the apparent organization of seam cells differs from that observed in *H. glycines.* The presence of phalloidin-stained structures similar to seam cell apical junctions in the subventral and subdorsal quadrants of *M. incognita* in post-infective stages could point to the formation of a separate seam cell pool. This will require further examination using both additional light microscopy and TEM. One obvious caveat to the phalloidin staining is its unspecific nature as a general F-actin probe. The unusual pattern of staining in *M. incognita* may not indicate apical junctions, but rather other actin-enriched structures. As *H. glycines* and *M. incognita* likely evolved saccate morphologies independently, we suggest that this serves as an example of parallel evolution where a similar parasitic environment led to selection for increased proliferation of the seam cells acting through distinct mechanisms.

The evolution from vermiform to saccate nematode may have occurred through an intermediate transitional stage as represented by *R. reniformis,* which shows no obvious epidermal proliferation following infection. The increase in *R. reniformis* body volume from vermiform to saccate is substantially smaller than that found in *H. glycines. H. glycines* produces more offspring than *R. reniformis* [27, 28], and it is generally accepted that saccate nematodes have a greater reproductive potential than vermiform species. We observed that the swelling of *R. reniformis* began proximal to the growing gonad. In contrast, the increase in *H. glycines* body volume far outpaces the development of its gonad (here and [29]). We speculate that the increased fecundity of saccate nematodes began with the evolution of a larger gonad size similar to *R. reniformis.* This was followed by proliferation of the epidermis allowing for larger gonads with additional reproductive capacity. This hypothesis will require extensive examination of saccate species from additional clades.

## Conclusions

Our data reveal that seam cell development has undergone extensive evolutionary changes from *C. elegans* and that the number and ploidy of *H. glycines* epidermal nuclei correlate with growth in size. Interestingly, the change in the number of *H. glycines* epidermal nuclei and ploidy level does not fit a previously established model of body volume in vermiform species, suggesting that these factors may regulate other aspects of development in the Tylenchomorpha species. Our finding of different seam cell division patterns in the independently evolved saccate species *M. incognita* and *H. glycines* provides an example of parallel evolution acting through homologous cells. The number of epidermal nuclei and ploidy level is associated with *H. glycines* and *M. incognita* shape changes. However, *R. reniformis* does not demonstrate extensive seam cell proliferation following infection suggesting distinct mechanisms evolved to produce a similar phenotype from a common ancestor. We hypothesize that *R. reniformis* may serve as an extant transitional model for the evolution of atypical body shapes.

## Materials and methods

### Nematode cultures

*Heterodera glycines* was isolated from a soybean field in Illinois, USA, and maintained on susceptible soybean (cv. Macon) in the greenhouse. To collect synchronized developmental stages of *H. glycines,* soybean seeds were germinated in moist paper towels for 3 days. Soybean seedlings were then placed in pluronic gel F-127 with freshly hatched J2 for 24 h [30]. Soybean roots were washed with water to remove nematodes that had not infected roots within 24 h of inoculation. Then, *H. glycines*-infested roots were planted in sandy loam soil and kept in a growth chamber at 22–24 °C and 12-h light cycle until extraction. After inoculation, the infested roots were grown for 6–7 days to collect J3 s, 8–10 days to collect J4 s, 11–13 days for adult females, and 15 days for adult males [31]. Infested roots were macerated with a hand blender to obtain *H. glycines* at specific time points. The mixture was poured over stacked 850-, 250-, and 25-μm-pore sieves. The developmental stage and sex of each individual were determined based on overall body size and gonad morphology [29].

*Meloidogyne incognita* (gift from Dr. Jason Bond) was originally isolated from soybean and maintained on tomato (cv. Rutgers) in the greenhouse. To collect synchronized developmental stages of *M. incognita*, two-week-old tomato seedlings were inoculated with freshly hatched J2 s. Tomato seedlings were gently removed from the soil and washed with water to remove nematodes that had not infected roots within 48 h following inoculation. *M. incognita*-infested roots were then planted in sandy loam soil and kept in a growth chamber at 22–24 °C and a 12-h light cycle until extraction. The nematodes were extracted using a hand blender to collect different developmental time points of post-infective J2 s [7].

*Rotylenchulus reniformis* (gift from Dr. Martin Wubben) was maintained in sandy loam soil on soybean (cv. Macon) in the greenhouse. To study the non-parasitic stages, eggs were incubated at 30 °C. Different developmental stages were identified based on their morphology and number of cuticles [4]. *R. reniformis* parasitic adult females were extracted as described above for *H. glycines*.

*Aphelenchus avenae* was originally isolated from the rhizosphere of garlic plants and identified using morphological characters. *A. avenae* was cultured on 1/8 strength Potato Dextrose Agar (PDA) with the fungus *Botrytis cinerea* [32]. To collect the synchronized *A. avenae* eggs, gravid females were incubated in 5% M9 buffer [33]. After 5 h, females were removed, and eggs were incubated at room temperature for approximately 48 h. Hatched J2 s were transferred to 1/8 strength PDA with *B. cinerea.* Developmental stages were determined based on body size and gonad morphology.

*Pratylenchus penetrans* (gift from Dr. Terry Niblack) was cultured on corn root explants on Murashige and Skoog (MS) media [34]. Specific developmental stages of *P. penetrans* were isolated by synchronizing populations from eggs. *P. penetrans* eggs were extracted as previously described [35]. Freshly hatched J2 s were collected and placed on corn root explants on MS media. To recover different developmental stages, *P. penetrans* culture plates were flooded with water and animals were handpicked under a dissecting scope. Developmental stages of *P. penetrans* were determined based on body size and gonad morphology.

*Helicotylenchus* sp. was isolated from soybeans at the University of Illinois research farm and maintained on soybean (cv. Macon) in sandy loam soil in the greenhouse. J4 molting stage animals were determined based on the presence of a shedding cuticle, body size, and gonad morphology.

### Live nematode imaging

Nematodes were mounted on a 4% agarose pad on microscope slides kept at room temperature in a dark humidity chamber when not imaging. Slides were rehydrated as needed. Images were taken every hour for 2 days, using an upright compound microscope with a mechanized stage (Zeiss M2 AxioImager and Zen software). Seam cells were identified based on their location and morphology [10].

### DAPI (4′, 6-diamidino-2-phenylindole) staining

*Heterodera glycines* was fixed in Carnoy’s fixative (60% ethanol, 30% acetic acid, 10% chloroform) overnight at room temperature [8, 17]. Nematodes were transferred to 75% ethanol and stained with 0.2–0.5 μg/ml of DAPI overnight in the dark at room temperature. Images were taken using a Zeiss M2 AxioImager with differential interference contrast (DIC) and fluorescence optics. In *C. elegans*, the epidermal nuclei are large and flat, contain large nucleoli and are located in four main cords [10]. The number of epidermal-like nuclei was counted from the metacarpus to anus. The number of epidermal nuclei counted on one side of the animal was doubled to estimate the total number of epidermal nuclei. To estimate the body volume of *H. glycines,* the length of the animal was measured from head to tail, and the mean diameter was calculated from three separate measurements (close to the head, the middle part of the body, and close to the tail) using FIJI. The volume was then estimated based on the equation for the volume of a cylinder during J2 and J3 and the shape of a sphere in adult females. At least six animals were examined for each developmental stage. The data were log transformed and regression analysis was performed using GraphPad Prism version 7.00 for Windows, GraphPad Software, La Jolla California USA, www.graphpad.com.

The ploidy of *H. glycines* epidermal nuclei was calculated based on previous methods [8, 17]. DAPI-stained sperm nuclei from adult males were used as a haploid control and included in each experimental session. Fluorescence intensity, exposure time, and all other microscope settings were kept consistent during imaging. The fluorescence level of the epidermal nuclei and sperm nuclei were measured using ImageJ software. The nuclei of interest were marked and their integrated density (fluorescence in the area of the region of interest × the mean fluorescence of the region of interest) was measured. The corrected fluorescence intensity was calculated by subtracting the background fluorescence in eight male animals, eight J2 molting stages animals, five J3 females, and 13 adult females [36]. In each animal, the fluorescence intensity of ten epidermal nuclei and ten neuronal nuclei was measured.

All other nematodes (*M. incognita, R. reniformis, A. avenae, P. penetrans,* and *Helicotylenchus* sp.) were fixed in Carnoy’s fixative for 2 h and then transferred to 50% methanol and stained with 0.2–0.5 μg/ml of DAPI overnight in the dark at room temperature until imaging. All images were captured with Zen software on a Zeiss M2 AxioImager with DIC and fluorescence optics and analyzed in FIJI. To examine the correlation between body volume and the number of epidermal nuclei among different nematode species, young adult females of *M. incognita* and *R. reniformis,* and J4 molting females of *A. avenae*, *P. penetrans* and *Helicotylenchus* sp. were examined. Nematode body shapes at these stages were considered to be cylindrical and the volume calculated as described for *H. glycines*. At least five animals were examined in each species. The data was log transformed. Regression analysis was performed using GraphPad Prism. Unlike Flemming et al. [8], we present the number of nuclei as the estimated total number rather than that found on one side of the body. We doubled the number of epidermal nuclei from Flemming et al. [8] to estimate the total number of nuclei in each species and reproduced the regression model based on their data. We mapped Tylenchomorpha nematode species on their regression model with 95% prediction intervals.

### Phalloidin staining

Nematodes were fixed in 4% paraformaldehyde overnight at 4 °C and washed three times with water. Nematodes were placed in a 3-mm Petri dish and cut open with a 1.2 mm × 25 mm BD precision glide needle (Becton, Dickinson, and Company). Nematodes were then transferred to phalloidin (5–7 unit/ml; Thermo Fisher Scientific) and DAPI (0.2–0.5 μg/ml; Thermo Fisher Scientific) and incubated overnight in the dark at room temperature. The images were taken using a confocal microscope (Zeiss LSM 880 Airyscan) and analyzed using ImageJ software.

### Electron microscopy

Synchronized developmental stages of *H. glycines* were collected from soybean roots and stored overnight at 4 °C. High-pressure freezing and freeze substitution were modified from previous methods used for *C. elegans* [37, 38]. Metal specimen carriers were coated with 1-hexadecene and a layer of *E. coli* strain OP50. Nematodes were loaded into carriers with 20% bovine serum albumin and frozen in an HPM 010 high-pressure freezer. Freeze substitution was performed with 2% OsO_4_ (Electron Microscopy Sciences), 0.1% uranyl acetate (Polysciences), and 2% H_2_O in acetone in an FS-8500 freeze substitution system. Samples were kept at − 90 °C for 110 h before being warmed to − 20 °C over 5 h. Samples were then kept at − 20 °C for 16 h before being warmed to 0 °C over 5 h. Samples were washed four times in pre-chilled 100% acetone at 0 °C. The last wash was 1 h. Samples were then transferred to room temperature and washed two times in 100% acetone. Samples were infiltrated with 1:1 Polybed812 (Polysciences) resin/acetone for 24 h, 2:1 resin/acetone for 36 h, 100% resin for 24 h, and then changed to fresh resin for 3 days. All infiltration steps were conducted on an orbital shaker at room temperature. Samples were then submerged into embedding molds with resin and hardener and baked at 60 °C for 2 days. 70 nm sections were collected using a PowerTome PC ultramicrotome with a diamond knife and collected onto formvar-coated copper slot grids. Sections were stained with lead citrate and uranyl acetate and imaged with a Phillips CM200 TEM [25].

## Abbreviations

DAPI: 4′, 6-diamidino-2-phenylindole
DIC: differential interference contrast
TEM: transmission electron microscopy
DPI: days post-infection
